# A Low Cost and Portable Microwave Imaging System for Breast Tumor Detection Using UWB Directional Antenna array

**DOI:** 10.1038/s41598-019-51620-z

**Published:** 2019-10-29

**Authors:** M. T. Islam, M. Z. Mahmud, M. Tarikul Islam, S. Kibria, M. Samsuzzaman

**Affiliations:** 1 Center of Advanced Electronic and Communication Engineering, Faculty of Engineering and Built Environment, Universiti Kebangsaan Malaysia, Bangi, 43600 Malaysia; 2 Department of Computer Science and Engineering, Jagannath University, Dhaka, Bangladesh

**Keywords:** Biomedical engineering, Electrical and electronic engineering

## Abstract

Globally, breast cancer is a major reason for female mortality. Due to the limitations of current clinical imaging, the researchers are encouraged to explore alternative and complementary tools to available techniques to detect the breast tumor in an earlier stage. This article outlines a new, portable, and low-cost microwave imaging (MWI) system using an iterative enhancing technique for breast imaging. A compact side slotted tapered slot antenna is designed for microwave imaging. The radiating fins of tapered slot antenna are modified by etching nine rectangular side slots. The irregular slots on the radiating fins enhance the electrical length as well as produce strong directive radiation due to the suppression of induced surface currents that radiate vertically at the outer edges of the radiating arms with end-fire direction. It has remarkable effects on efficiency and gain. With the addition of slots, the side-lobe levels are reduced, the gain of the main-lobe is increased and corrects the squint effects simultaneously, thus improving the characteristics of the radiation. For experimental validation, a heterogeneous breast phantom was developed that contains dielectric properties identical to real breast tissues with the inclusion of tumors. An alternative PC controlled and microcontroller-based mechanical MWI system is designed and developed to collect the antenna scattering signal. The radiated backscattered signals from the targeted area of the human body are analyzed to reveal the changes in dielectric properties in tissues. The dielectric constants of tumorous cells are higher than that of normal tissues due to their higher water content. The remarkable deviation of the scattered field is processed by using newly proposed Iteratively Corrected Delay and Sum (IC-DAS) algorithm and the reconstruction of the image of the phantom interior is done. The developed UWB (Ultra-Wideband) antenna based MWI has been able to perform the detection of tumorous cells in breast phantom that can pave the way to saving lives.

## Introduction

Worldwide, breast cancer is reported to be the leading cause of women’s death. Every year more than 1.8 million new cases of breast cancer are spotted. Breast cancer is caused due to the presence of malignant or tumorous cells in the breast tissues^1^. It is not an incurable disease. Reliable diagnosis in an earlier stage is the key factor in treating breast cancer. With early detection, treatment of breast cancer can reach a survival rate of up to 97%, emphasizing the urgent need for a highly efficient and reliable technique to detect the earlier breast cancer^2^. X-ray mammography, computed tomography (CT) scan, Ultra-Sonic imaging (US), magnetic resonance imaging (MRI) are often used to detect breast cancer^3,4^. These techniques have some limitations such as false positive and missed detection, low-resolution scan, higher-cost, uncomfortable compression and ionization, performance degradation for the deep-lying or solid tumor and time-consuming process of diagnosis.

In literature^5^, a study is carried out with 258 patients, where 177 had benign tumors and 177 having malignant tumors. The metrics used to test the current detection techniques were sensitivity, specificity, positive predictive value, and accuracy. The performance of various detection technique combinations was also compared^5,6^. The maximum sensitivity is achieved by combining the MRI, X-ray mammography, and a few other clinical approaches. The best detection performance was about 94.4%. The highest rate of accuracy is obtained about 75.6%, which proves that one-fourth of diagnoses are false positives. This requires an efficient, low-cost, non-ionizing, portable, and comfortable technique as a complementary tool to the clinical approaches currently in use^7^. MWI and THz system are now becoming a hot research topic for both as an alternative solution as well as a complementary tool to the available techniques. Researchers are trying to overcome the limitations in terms of high-cost, false detections, low-resolution images, and tedious measurement process. The THz spectrum lies between Infrared (IR) and Microwave, and it finds many applications in Biology, medicine, Imaging, Security, etc. Terahertz radiation is electromagnetic waves in the spectral range going from 0.3 to 10 THz^8^. The use of THz frequency is evolving gradually, and the need for research in developing a THz based cancer detection system is increasing^9^. THz radiation has some attractive features like it can penetrate through a wide variety of dielectric materials, non-ionizing radiation and has minimal effects on the human body, diagnostic capabilities, large absorption due to water, Metals are highly reflective in the terahertz bands. Despite numerous advantages, imaging with a THz presents some limitations like the small generated power (less than a dozen μW average power), detecting terahertz signals is difficult and complex because blackbody radiation at room temperatures is strong at terahertz frequencies, no efficient 2D coherent detectors are available, and image acquisition rate is slow^10^. In comparison, THz to microwave imaging, the THz imaging is more complex, expensive, coherent detection harder at a higher frequency and more ionization effect. The advantages of MWI are higher positive results, lower in cost, higher data rate, low complexity, convenient and low-power density.

Meaney *et al*.^11^ presented an experimental prototype of near field MWI at Dartmouth College. The system uses sixteen transceiving monopole antennas within the frequency range of 300–1000 MHz. A reservoir tank was used between the antennas and the human breast as a coupling media. By using the mechanical switch, the array of antennas were moved vertically and tuned to the chest level. Most of the above-mentioned antennas and microwave systems used synthetic materials and matching liquid, which has very close electromagnetic properties to the genuine breast tissues. A UWB antenna based MWI system along with experimental results, is presented in literature^12^. For detection of breast tumor, an artificial breast phantom and a hemispheric array of the stack-patch antenna was used. Although there was no requirement of mechanical scanning, this technique was reported as the first-ever use of real-time radar-based measurement. The proposed system exhibits some limitations in terms of cluster rejection and resolution. A two-dimensional configuration was developed to identify the pathological functions of soft tissue^13^. The system has been tested to define the soft tissues pathological functions and intervention. The system was constructed with a two-dimensional imaging chamber consisting of a single metallic cylinder and twenty-four antennas equidistantly located in the horizontal cross-section. Salt, alcohol, and intralipid emulsion are mixed to fill up the chamber. By using a control unit, a single antenna is used for electromagnetic transmission, and 23 others are used for measuring the electromagnetic field. In^14^ an iterative method was proposed to present the time differential images by using a 2D born algorithm and 2D Newton method. Appropriate electrical and mechanical combinations were used for a pilot clinical trial in^15^. Monostatic mechanical scanning system was developed by using antipodal Vivaldi antenna (AVA). A laser and a cylindrical tank were combined with canola oil as coupling liquid. The obtained results were consistent by using the delay-and-sum algorithm within the range of 0.05 to 15 GHz. In^16^ a 16-element antenna array-based time-domain radar system was designed over the 2 to 4 GHz frequency range. A hemisphere radome is used in the system as a phantom. Porter *et al*. present a clinical trial system with repetitive measurement capability and reconstructions of images. A pulse-shaping circuit, directional coupler, reflector, and amplifier were used to design the time domain radar.

Amineh *et al*. proposed a UWB antenna based near field microwave imaging system with raster scan algorithm^17^. Both measured and simulated results were presented by the researcher using a three dimensional (3D) homogeneous breast phantom and a heterogeneous model. The proposed transverse electromagnetic (TEM) horn antenna is directive in nature, which helped the scanning system to obtain stronger scattering signal from the dielectric phantom. The antennas of the proposed system were directly contacted to the imaging domain. Flores *et al*. investigated a preclinical experimental prototype using single element Vivaldi antenna to study the cylindrical-shaped dielectric objects. To measure the reflection coefficient (S_11_), a canola oil filled plexiglass tank is used as the imaging domain. Circular scanning geometry and the phantom rotating platform is used to improve the qualitative and quantitative images. In another study^18^, a Finite Difference Time Domain (FDTD) algorithm based tumor detection technique is presented. A cylindrical tank full with water was utilized as the imaging domain, and low permittivity iron rod was used as a tumor. The value of permittivity and loss of water are much higher compared the to actual breast tissue and air, which made higher reflection and attenuation at air-skin interface compared to the materials employed in the experiment. Mohammed *et al*. Presented a 12 element TSA planner antenna based microwave imaging system at the University of Queensland^19^. A suitable imaging platform was considered with the combination of VNA (Vector Network Analyzer), SP6T (Single Pole Six Throw) switch, artificial breast phantom, and intermediary coupling liquid. The mutual-coupling was observed by the trust-region framework, and the proposed system was verified for a breast imaging application. Though the preparation of testing platform is presented but no signal and image processing mechanism is provided. The system was limited only with mutual coupling between antenna pairs. A Vivaldi antenna based breast tumor detection system is presented by Zhang *et al*.^20^. The system uses commercially off-the-shelf breast phantom and cooking oil. The transmitted pulses were received by sampling oscilloscope. The background subtraction algorithm was used to collect the reflection data from the dielectric targets.

Though the microstrip antennas are quite simpler in design and cost-effectiveness, it has some limitations like lower gain (<5 dBi) and radiation performance. To overcome the limitations, numerous techniques are presented by researchers. The techniques included the use of unit cells^21^, cross-sectional Vivaldi antennas^22^, metamaterial (MTM) antennas^23^, slotted antennas^24^, use of an electromagnetic structure^25^, artificial magnetic conductor (AMC)^26^ and many more. These techniques are applied to make the UWB antennas compatible with microwave imaging applications. The Vivaldi antenna is one of the valuable tools for MWI due to its attractive features like compact size, broader bandwidth, higher directivity, lower in cost, and end-fire a radiation^27^. The main challenges of designing a Vivaldi antenna are to obtain the desired performance at lower frequencies as well as to maintain directive radiation pattern while retaining the compact size. Vivaldi antennas have been the subject of research in medical applications for the last few years^27^.

Academics over the globe have presented different techniques to improve Vivaldi antennas performance^28^. For improving the performance of Vivaldi antenna, Nasser *et al*.^29^ proposed a method by adding an ellipse-shaped parasitic element on the flare. The parasitic element increases the field coupling; whereas, it does not compromise the antenna size (140 × 66 mm^2^) and does not reach lower frequencies. A cavity-backed Vivaldi antenna (CBVA) for breast tumor detection system is proposed in^30^. The use of (CBVA) antenna size is reduced significantly but fails to reach the higher gain. In^31^ a tapered slot (TSA) Vivaldi antenna with square shape (75 × 75 mm^2^) is investigated. In this study, the antenna parameters are optimized to obtain the directive radiation patterns; however, it missed the resonance at a higher frequency. To enhance the gain and directivity, a customized Vivaldi antenna is fabricated with planner directors^32^. The antenna dimension is increased significantly (110 × 260 mm^2^), and it maintains a noncontagious VSWR. A compact sized metallic bending feed line structure based antipodal Vivaldi antenna (AVA) with modulated Gaussian slots is also reported in literature^33^. It has a fractional bandwidth with wider working frequency; however, the reflection efficiency (70%) and gain (<5 dBi) are not adequate. For the medical imaging applications, several antennas were recommended with different shapes and sizes, gain and efficiencies, resonance, and operating frequencies^34–36^. In another study^19^, a planner antenna array of 12 corrugated TSA was developed for the use of UWB biomedical MWI with moderate gain and low profile. Though the performance is fairly good but it achieved with the expense of antenna size and higher dielectric constant. The starting operating frequency is 5 GHz wthe hich is quite high. In the works presented in literature^37,38^, the polar Vivaldi antennas are presented by considering the bandwidth, gain and impulse response. These presentations are costly in terms of antenna size, where antenna dimension should be compact.

This work presents a complete, low cost and portable microwave imaging system (MIS) for the detection of unwanted tumor cells inside the human breast. Firstly, side slotted tapered slot UWB antennas are designed, developed, and fabricated, which satisfied all the requirements for MIS application. A novel miniaturization technique is proposed to minimize the antenna size. This approach enhances the operational frequency band and improves the efficiency and gain with miniaturizing the antenna size of about 49%. The proposed antennas are found to be efficient in terms of reflection coefficient, gain, efficiency, radiation pattern, current distribution, and both in frequency and time domain characterizations. Secondly, experimental validations of the antennas have been performed by designing and developing an MIS system to detect the tumors inside the breast phantom. Heterogeneous breast phantom with tumor inclusion has been developed. The developed phantom poses dielectric properties similar to the real breast tissue. The backscattered data are collected and processed using the newly proposed IC-DAS imaging algorithm to reconstructs the breast interior to detect the tumor using Matlab. The variation of the backscattered signal can exploit the tumor cells into the breast, which have higher dielectric values than the normal breast tissue. Finally, the developed UWB antenna based MIS has been able to perform the detection of tumorous clusters in breast phantoms, which potentially makes it viable for clinical trials.

## Methodology

The microwave imaging technology is based on the different dielectric properties of the tumor and the surrounding healthy tissues. In terms of microwave frequencies, the tumor and healthy tissues reflect microwave differently, which is the principle to apply to the practical system for detecting cancerous cells. An antenna array will be used to send microwave pulses to the suspected area of human tissue. The signals are reflected through backscattering picked up by the array and then analyzed using a suitable computing system to detect if there is a tumor present^39^. In order to obtain high resolution and accurate images, the compact antennas must be able to radiate signals over a broad range of frequencies while maintaining the fidelity of the waveform over a wide angular range in such microwave imaging systems using the UWB^40^. One of the possible option to meet the requirements is the planner structure printed circuit board on which the antenna can be printed^41^.

## Phantom Materials and Methods of Preparation

**Figure 1:**
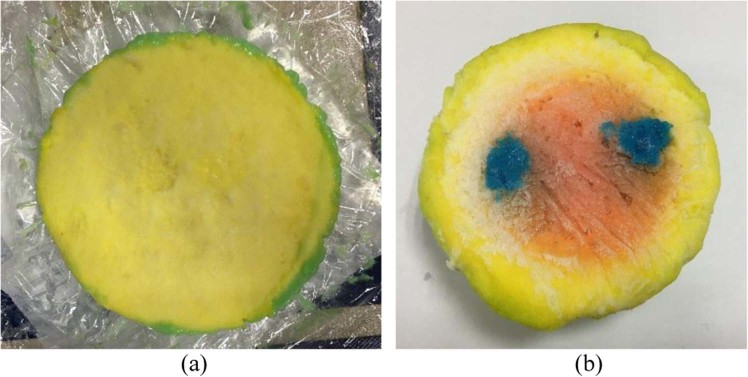
Developed heterogeneous breast phantom (**a**) without tumor (**b**) with two tumors.

In the construction of a breast phantom, four different layers were considered, these are; skin, gland, fat, and tumor. The phantom is fabricated and measured based on the procedures depicted in literature^42^. The dielectric properties of numerous tissues are considered by permittivity, which is the mean of complex-valued dielectric,

1 $$\varepsilon (\varepsilon ={\varepsilon }_{r}+i\sigma /\omega {\varepsilon }_{0})$$

where *ε*_*r*_ represents dielectric constant and *σ* denotes the conductivity of the tissue against frequency. The dielectric permittivity of vacuum here is *ε*_0_, and the angular frequency is *ω*. The image of the fabricated heterogeneous phantom is illustrated in Fig. 1.

## Microwave Imaging System

**Figure 2:**
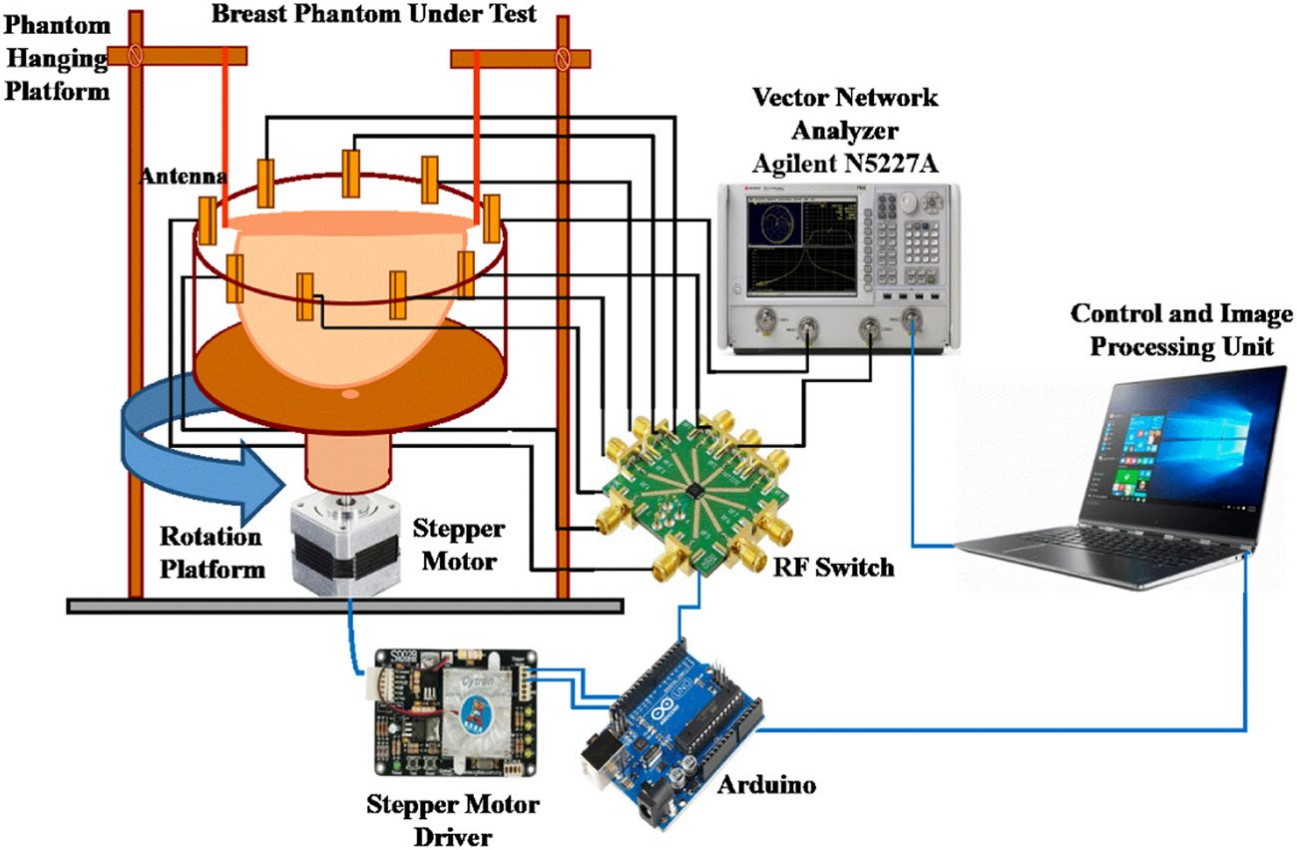
The diagram and different components of the proposed breast imaging system.

**Figure 3:**
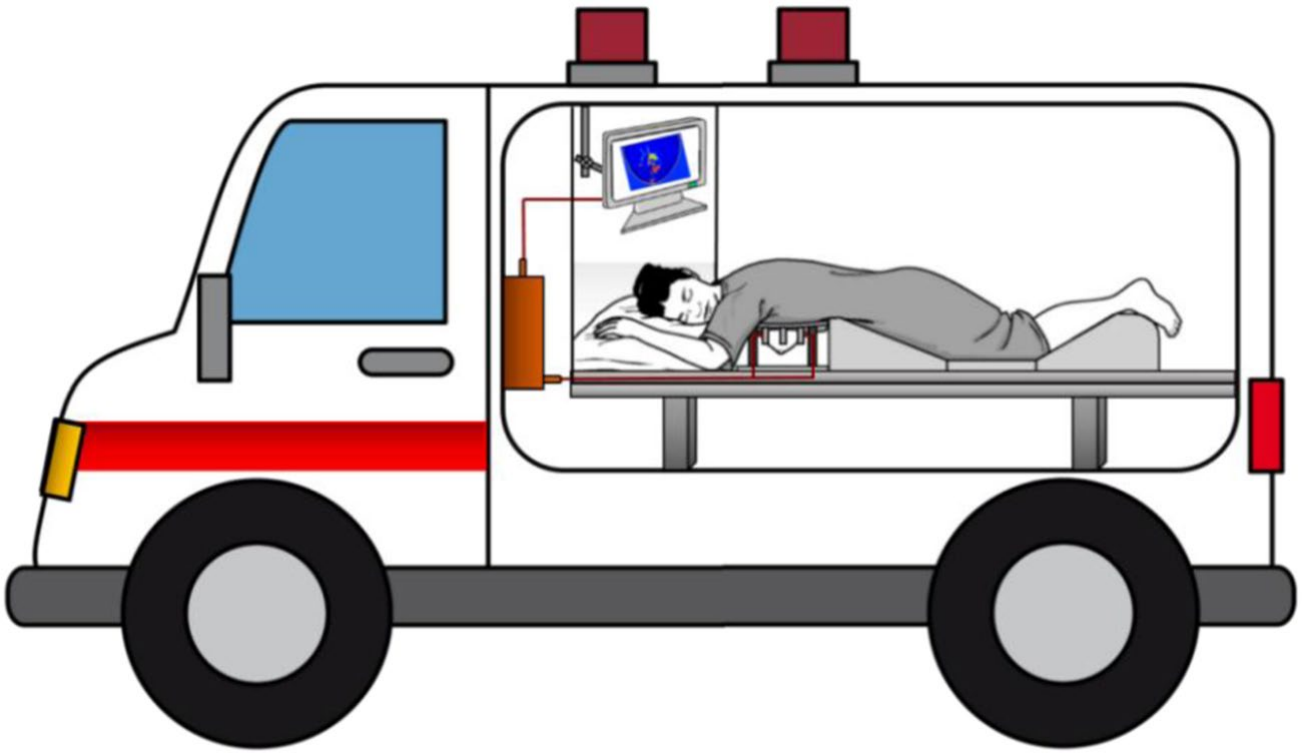
The graphic view of the proposed portable breast imaging system.

The architecture and different component of the proposed imaging system are depicted in Fig. 2. The designed MIS consists of nine antenna array, where one antenna works as transmitter and the other eight antennas receive the scattered signals, the stepper motor based antenna mounting stand, the adjustable phantom hanging platform, a 9 port RF switching system to control the receivers and a MATLAB based laptop computer program for signal processing and image reconstruction. The antennas are set up on an adjustable transparent circular ABS material based container. The plastic container placed on an SD02B controlled Stepper Motor. The phantom is placed inside the antenna array using the hanging platform and scanned using the antenna array. The gap between the mounted antenna and the phantom is maintained at 2 cm. The mechanical rotation platform can rotate the array in polar coordinates from 0 to 2π around the breast phantom using the stepper motor. The antennas are connected to the RF switch using coaxial cables. The port 1 of VNA generates microwave signals in the frequency domain and transmits it to the phantom. The received backscattered signal from eight receivers antennas is collected by switching the receiving antennas through VNA (Agilent E8358A) and MATLAB program in PC. The backscattered data (S_21_, S_31,_ S_41_,….S_81_) are collected in each 7.2° rotation where the total 360° is covered by 50 equal points. The graphic view of the proposed portable system is presented in Fig. 3.

**Figure 4:**
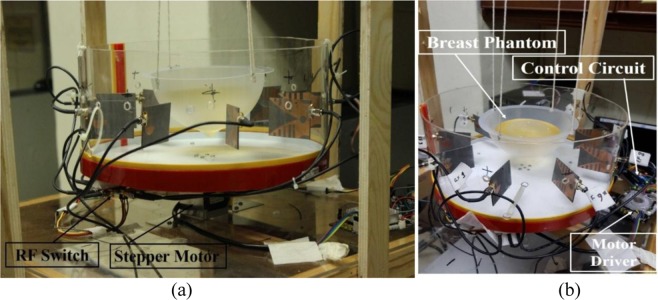
Imaging domain (**a**) Side view (**b**) Top view.

All these devices and electromechanical circuits related to the data acquisition process are controlled by a PC controlled Arduino Uno system which is connected to the personal computer through the USB port. The collected data are processed using an imaging algorithm which reconstructs the image of the breast interior to detect the tumor. The imaging domain of the proposed system is shown in Fig. 4. In this research, no human body or patient due to the safety and ethical considerations. The breast phantom is placed inside the antenna array, and the attenuation and reflection of microwave signal due to the breast tissues are considered. To alleviate air-interference, the entire imaging system is calibrated across the operating frequency using the Agilent 85052D calibration kit.

## SSVA Design and Performance

**Figure 5:**
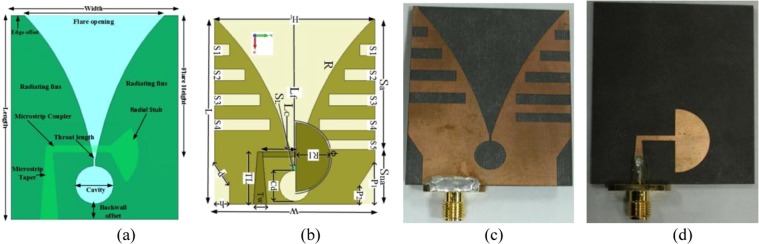
Fundamental geometry (**a**) exponentially tapered slot (**b**) proposed modified antenna (**c**) front view of fabricated prototype (**d**) back view of fabricated prototype.

Vivaldi is one kind of traveling wave antenna whose current is the same amplitude but different phases. Vivaldi antennas boast highly directive radiation with high gain because of its tapered slot design and the peak value of is high due to the pulse envelope. The substrate used in the antenna should be considered in terms of both the dielectric constant, which shrinks the antenna dimension and the thickness which controls the gain and main beam-width. The trade-off between dielectric constant, dimensions, and bandwidth needs careful consideration. To attain the required characteristics stated earlier, Vivaldi antenna had been customized to side-slotted (SSVA)^38,43^. The fundamental geometric layout of an exponentially tapered slot Vivaldi antenna and the proposed antenna geometry is presented in Fig. 5(a,b). Figure 5(c,d) shows the fabricated antenna prototype. The modification of the structural design of stub angle, cavity, and radiating fins help to achieve the compactness of the electrical dimension with a higher gain. In this work, Rogers RT/duroid 5870 substrate is used. The height of the substrate is 1.57 mm having 35 μm copper on both sides. The relative permittivity and the loss tangent is 2.33 and 0.0012. The overall dimension of the antenna is 51 × 42 mm^2^. The SSVA’s radiation properties are determined by several exponential curves, slot lines, cavity structure, the arrangement of the stub, back wall offset, tapered rate, position feedline, and the structures of the radiating fin. In the proposed structure, all of these parameters are adapted.

The main radiating element of the proposed antenna is determined by an exponential opening slot line known as a flare. The height of flare (*H*_*f*_) is 41 mm, and the length of flare (*L*_*f*_) is 38 mm with the value of tapered rate *R* = 0.04. The exponential tapered slot line is also known as radiation section since the wave is radiated along the slot line. The below exponential curve equation is used to design the proposed Vivaldi antenna as:

2 $$x={C}_{1}{e}^{Rz}+{C}_{2}$$

where $${C}_{1}=\frac{{x}_{2}-{x}_{1}}{({e}^{R{z}_{2}}-{e}^{R{z}_{1}})}$$ and $${C}_{2}=\frac{{x}_{1}{e}^{R{z}_{2}}-{x}_{2}{e}^{R{z}_{1}}}{({e}^{R{z}_{2}}-{e}^{R{z}_{1}})}$$

The points (x_1_, z_1_) and (x_2_, z_2_) are the endpoints of the flare. The maximum open size of flare exponential curves slot lines is the flare opening or mouth opening. The narrowest part of the curve is exciting to point, and radiation propagates through the increasing of the curve. The flare opening should be larger than half wavelength, and with the increasing of flare opening the lowest working frequency is decreased.

At the end of flare there is a cavity with diameter, C_d_, of 8 mm, which is adjusted with the following equation:

3 $${C}_{a}=0.5{C}_{d}-0.5{C}_{d}(\cos \,\theta )$$

where, $\theta =\,\sin (\frac{{S}_{w}}{{C}_{d}})$, *C*_*d*_ is the cavity diameter.

4 $${C}_{a}=0.5{C}_{d}-0.5{C}_{d}(\cos \,\theta )$$

where $$\theta =\,\sin (\frac{{S}_{w}}{{C}_{d}})$$

The radiating properties of Vivaldi antennas are also dependant on the structure of the cavity, the arrangements of the stub, the slot line, the position of feedline, the tapered rate, the offset of the back wall, and the radiating fins’ structure. The cut-off or resonance frequency of the Vivaldi antenna also can be manipulated using the following equation^44^:

5 $${f}_{c}=\frac{c}{[w^{\prime} \sqrt{({\varepsilon }_{r}})]}$$

where *c* is light velocity in free space, *f*_*c*_ is the cut-off frequency, *ε*_*r*_ is the substrate’s relative permittivity and width of flare edge opening is $w^{\prime} .$

The antenna impedance is mainly controlled by the microstrip coupling line. When the thickness of the substrate is more smaller than the wavelength (h ≪ λ), the impedance characteristics can be calculated by:

6 $${Z}_{0}=\{\begin{array}{c}\frac{60}{\sqrt{{\varepsilon }_{e}}}\,\mathrm{ln}(\frac{8h}{{w}_{m}}+\frac{{w}_{m}}{4h}),\,\frac{{w}_{m}}{h}\le 1\\ \frac{120\pi }{\sqrt{{\varepsilon }_{e}}[{w}_{m}/h+1.393+0.667\,\mathrm{ln}({w}_{m}/h+1.444)]},\,\frac{{w}_{m}}{h} > 1\end{array}$$

where w_m_ indicates the width of the microstrip coupler line, and h is the substrate thickness.

**Figure 6:**
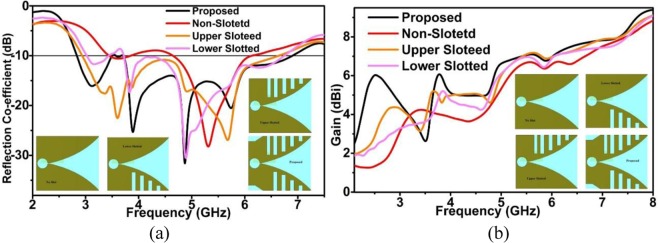
Effect of irregular slots (**a**) To the reflection coefficient and (**b**) To the gain.

**Figure 7:**
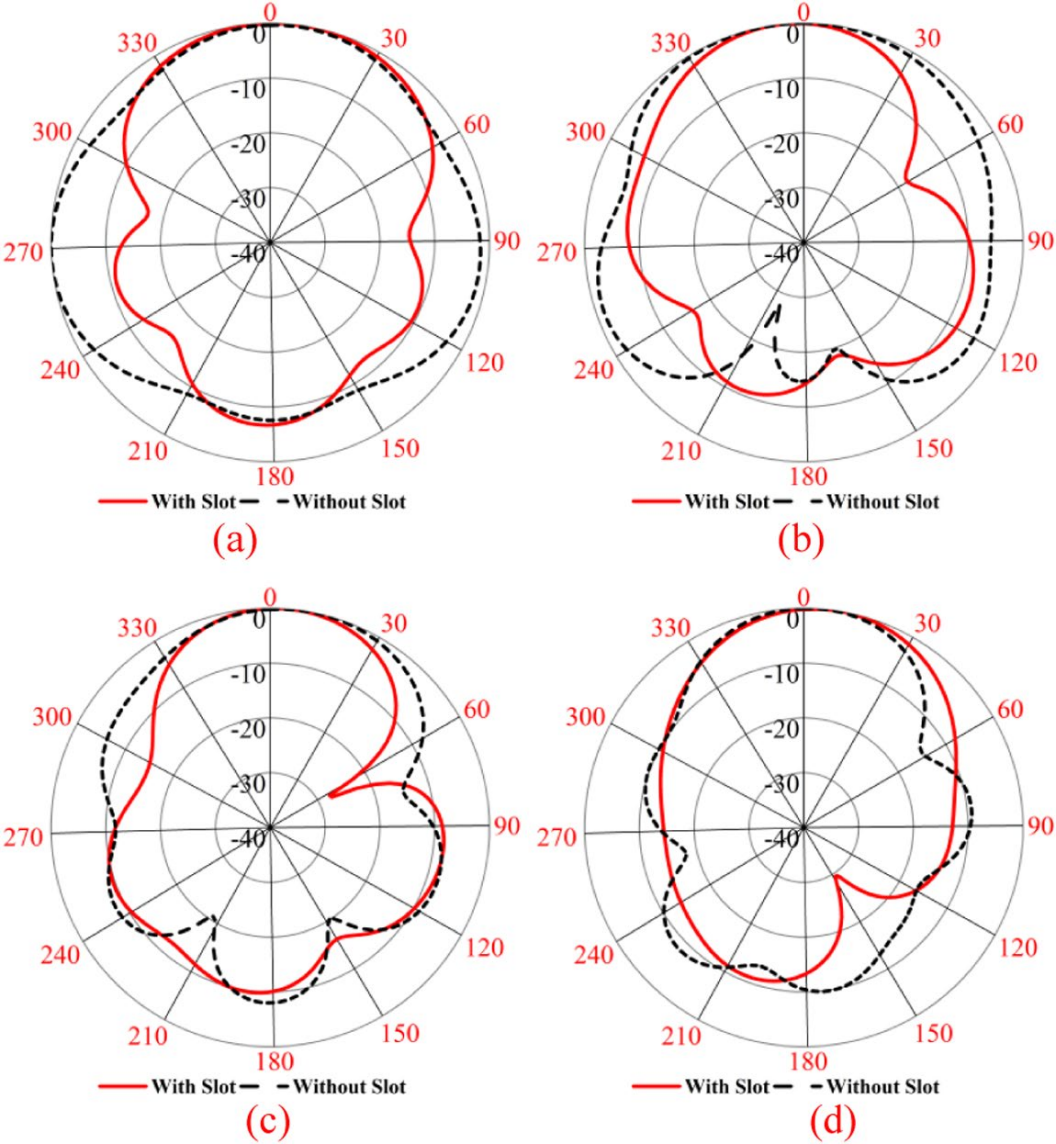
Radiation Pattern of xy-plane (**a**) 3 GHz (**b**) 3.75 GHz (**c**) 4.77 GHz (**d**) 5.78 GHz.

The whole conductor area of proposed SSVA is separated into two regions. These are slotted (S_a_) area and non-slotted (S_na_) area. Four identical rectangular slots (S_1_, S_2,_ S_3_, S_4_) are placed in both the bottom and topside. There is an extra slot, S_5_, at the top right side to enhance the efficiency of this extra slot. About 3% of efficiency is improved due to this extra slot. The size of rectangular slots is 4 × 3 (S_1_), 8 × 3 (S_2_), 12 × 3 (S_3_), 14.5 × 3 (S_4_), and 14.5 × 2.5 (S_1_). The gap between each slot in x-axis is 3.5 mm. Figure 6(a) displays the effect of slots on the reflection coefficient and gain. Reflection coefficient (S_11_) without slots is 3.40–6.08 GHz, with lower slots reflection coefficient (S_11_), is 3.04–6.70 GHz, with upper slots, S_11_ is 3.00–6.70 GHz and for all slots (proposed) S_11_ is 2.80–7.00 GHz with less than −10 dB. The reason for using these irregular slots is to obtain a higher electrical length as well as to obtain the lower operating frequency. Due to the presence of slots, the current path is changed and creates the higher-order current mode, resulting in more directionality. By adding slots, simultaneously, the sidelobe levels are reduced, the gain in the main lobe is amplified and the squint effect is corrected, providing an enhancement in radiation characteristics as shown in Fig. 7. The slots promote the optimal conditions for the main radiation lobe to contain maximum radiated energy. The slot edges control the current distribution around the lateral antenna edges which improves the antenna radiation characteristics. The side edge secondary current distribution leads to more efficient radiation in the end-fire direction across the operating band. The gain of the proposed antenna is better than the non-slotted or with upper and lower slot presented in Fig. 6(b). The slots on the radiating fins suppressed the unwanted surface current at the outer edges which flown vertically to the end-fire direction. It produces stronger directive radiation and increases the electrical length. The ratio of the slotted and non-slotted area is 62:32, which presents better performance. At the end of microstrip coupler line, there is a radial stub which reflects the incident energy to the microstrip coupling line. The stub angle and radius have a significant effect on the working frequency and the variation of radius and angle tune the impedance and bandwidth. The SSVA can be feed directly and electromagnetically. In this work, microstrip feeding is chosen due to its excellent performance in terms of bandwidth and beamwidth. A 50 Ω SMA connector with 2.08 dielectric constant and 0.000462 S/m conductivity is used to feed the antenna connected to microstrip tapered line. The parameters of the proposed SSVA are summarized in Table 1.

**Table 1:** Optimized parameters of proposed SSVA (in mm).

Parameters	Value	Parameters	Value	Parameters	Value
Length (L)	51	Opening rate (R)	0.04	Coupler width(W_c_)	1.4
Width (W)	42	Stub Radius (R_1_)	9	Coupler Length (L_c_)	9
Flare Height (H_f_)	41	Stub angle (ϕ)(deg)	180	Slot-line length (S_L_)	1.5
Flare length (L_f_)	38	Cavity Diameter(C_d_)	8	Quadrilaterals outer(P_1_)	11.15
Slotted area(S_a_)	36	Cavity Distance(C_0_)	0.76	Quadrilaterals inner(P_2_)	5.07
No slotted area(S_na_)	15	Tapered Width(T_w_)	3.5	Quadrilaterals height(h)	4
Slot-line width (S_w_)	0.5	Tapered Length (T_L_)	13	Quadrilaterals cross (d)	6.08

## Image Processing

**Figure 8:**
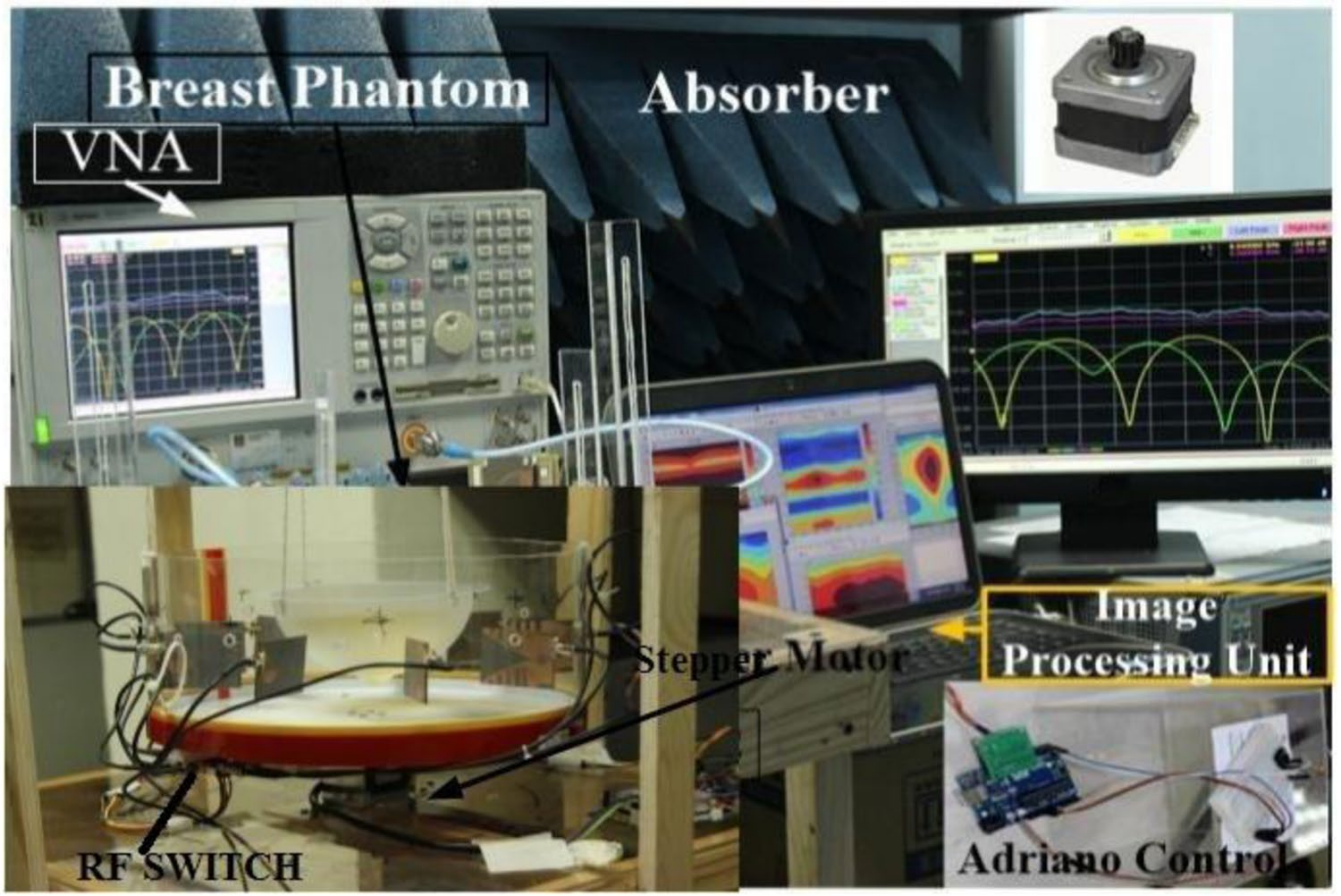
Experimental setup of the imaging system.

The primary goal of this system is to determine the change of the backscattered signal with the presence of a tumor. The heterogeneous breast phantoms are applied with a tumor of high dielectric constant. One breast phantom is with a single target, and another one is multiple targets. An automated microwave imaging system was designed for experimental validation. The breast phantom is placed on the rotating platform within the antennas. Figure 8 presents the proposed experimental setup of MWI system. The parameters of VNA are set as the IF-bandwidth is 10 Hz, the output power is 10 dBm, and the frequency range is 3.10 to 7.60 GHz. The microwave pulse is transmitted to the phantom from the transmitting antenna, simultaneously the reflected backscattered signals are collected by the receiving antennas. The transmission parameters depend entirely on the antenna path. Most of the reflected parameters present the shallow depths under the skin layer. The signals are bounced off to the opposite side of phantom and attenuated significantly. The reflected signals can perfectly be detected by the antenna which has higher gain, directional radiation pattern, and lower reflection coefficient.

## Image Reconstruction Algorithm

The method of removing the internal and external reflections of the skin from the scattered signal is critical. One of the preferred methods is rotation subtraction that relies on the contrast between the original signal and at least a single illumination of rotation^45^. In such systems, the offset data is collected using an array of antenna surrounding the scanning area. This experiment divided the matrices into odd and even where *φ* being odd or even, and the complete S parameter is represented as *S (f*, *Rx*, *φ*). The *φ*_*odd*_  = 1, 3, 5, …, N_*φ*_ − 1, and *φ*_*even*_ = 2, 4, 6, …, N_*φ*_ for the proposed setup. In this context, we consider the actual signal as *S*_*odd*_ and offset signal as *S*_*even*_. Thus we simply calculate the difference between these two matrices to implement rotation subtraction.

7 $${{\rm{S}}}_{\mathrm{skin}\_\mathrm{removed}}(f,Rx,{\phi }_{odd})={{\rm{S}}}_{{\rm{odd}}}(f,Rx,{\phi }_{odd})-{{\rm{S}}}_{{\rm{even}}}(f,Rx,{\phi }_{even})$$

Finally, the Inverse Fourier Transform is used to convert the signals to time domain *Γ(t*, *Rx*, *φ*_*odd*_*)*.

Basically DAS based algorithms consider a single target from where the reflections may occur. It becomes unstable when it is multiple object scenario as there is an internal reflection that can misguide the system. Iteratively corrected coherence factor delay and sum (CF-DAS) algorithm was introduced for breast phantom imaging^46^. Thus, IC-DAS is introduced in the proposed imaging system as it compensates the maximum number of iterations until convergence. This magnitude is used for scaling the results achieved from typical DAS.

### DAS

The 3D Cartesian coordinates of the single point are represented in three matrices, C, and a total number of the point *i*. The *i* by *i* matrices here is *P*_*C-C*_ and generated from C. The *A*_*Tx*_ and *A*_*Rx*_ matrices holds the 3D coordinates of the transmitter and receiver, individually. Total N/2 (N = 50) orientation is generated as we use 64 separate channel on each rotation, and each of them should be addressed during rotation subtraction. Being static, in the imaging system, the antennas changes their distance from the target point while rotating. In a stable situation, the *A*_*Txφodd*_ and *A*_*Rxφodd*_ are achieved. Then, from C, *A*_*Txφodd*,_ and *A*_*Rxφodd*_ the *P*_*Txφodd-C*_ and *P*_*C-Rxφodd*_ are evaluated that contains the space from the transmitter and receiver. Finally, the appropriate delay is calculated by dividing the total distance by the speed of light.

8 $$\tau (i,rx,{\phi }_{odd})=\frac{\sqrt{{\varepsilon }_{b}}({P}_{Tx{\phi }_{odd}-C}(i,tx,l)+{p}_{C-Rx{\phi }_{odd}}(i,rx,l))}{c}$$

where the dielectric constant of the contextual medium is denoted by *ε*_*b*_. This method ignores the presence of multiple reflections or tumor responses. Here we get the scattering intensity map (*ϒ*_*DAS*_*(i)*) by analyzing the correction among the delayed signals. The correction is calculated for typical DAS as follows:

9 $${\varUpsilon }_{DAS}(i)={({\int }_{-\infty }^{\infty }{\sum }_{\forall {\phi }_{odd}}{\sum }_{\forall rx}\Gamma (t-\frac{\tau (i,rx,{\phi }_{odd})}{\Delta t},rx,{\phi }_{odd})dt)}^{2}$$

where Δt represents the time step. The typical DAS is affected by the artifacts most of the time as it relies on the coherent outline of delayed signals in a near field scenario. We introduce a new iterative variant of the DAS algorithm to overcome these issues. Moreover, The IC-DAS is easy to implement as it is free from, relatively more complex calculations like curve fitting used in IDAS^47^.

### Delay estimation correction

The resulting time delay must be higher as the dielectric materials reduce the signal propagation speed. So, a higher value of *ϒ* can be referred to in a region of C, as the region contains a higher dielectric. By suitably increasing the distances in *τ* calculations, the additional time can be adjusted. To determine the most fitted delay and scattering intensity map valuation, an iterative method is introduced since the adjustments of τ render to an enhanced estimation of the map. However, the explicit use of *ϒ* can lead the process more vulnerable and sensitive to noise levels. For solution, a distance inverse weighted integral averaging is contemplated for the reconstruction of leveled map *ϒ* ′*(i)*.

10 $$\varUpsilon ^{\prime} (i)={\int }_{C}\frac{{\varUpsilon }_{DAS}^{n-1}(i)}{1+{p}_{C-C}(i,j)}dj$$

Then, we calculate the modified delay using the below equation:

11 $$\tau ^{\prime} (i,rx,{\phi }_{odd})=\tau (i,rx,{\phi }_{odd})+\frac{\varUpsilon ^{\prime} (i)}{c}$$

12 $${\varUpsilon }_{DAS}^{n}(i)={\int }_{-\infty }^{\infty }{\sum }_{\forall l}{\sum }_{\forall rx}S(rx,{\phi }_{l},{t}_{k}-\frac{\tau ^{\prime} (i,rx,l)}{\Delta t})dt$$

The map is reconstructed based on the new set of delays. Finally, the closure criterion checks for merging. Equations 10–12 are iteratively assessed for n = 1, 2 …. 7.

13 $${E}_{\varUpsilon }={\sum }_{\forall i}|{\varUpsilon }_{DAS}^{n}-{\varUpsilon }_{DAS}^{n-1}|$$

The iterative process is ended when E_*ϒ*_ lessens to the desired level of accuracy as convergence has already been accomplished. In this work, E_*ϒ*_10^−5^ is considered.

**Figure 9:**
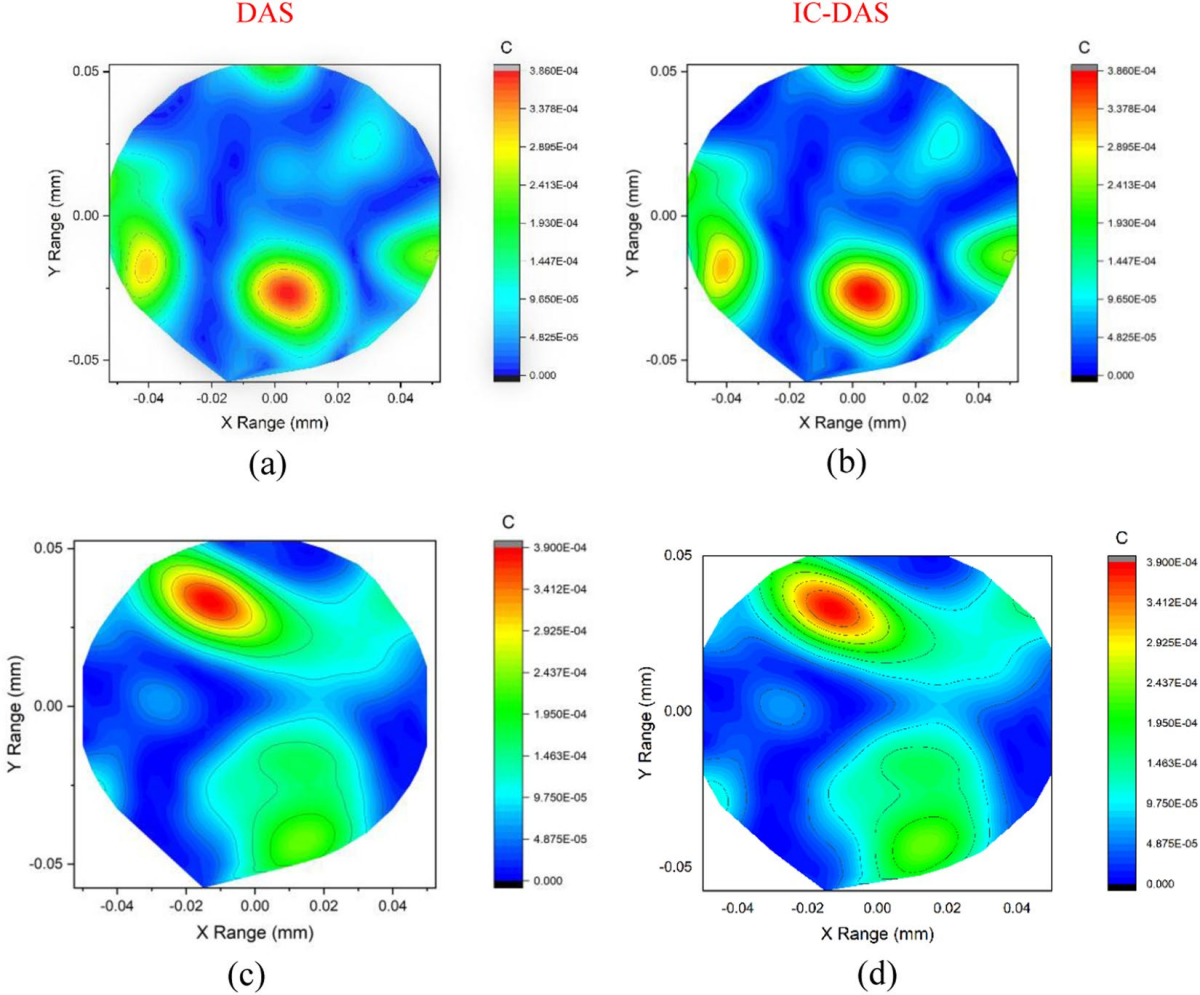
Reconstructed images for DAS (Left) and Proposed IC-DAS (Right) algorithm for two different phantoms (**a**) Phantom with a single tumor and (**b**) Phantom having two tumors.

The imaging performance of proposed MWI system using SSVA is shown in Fig. 9(a–d). Tumor detection for heterogeneous breast phantom is presented for both the conventional DAS and proposed IC-DAS algorithm. It is observed that by using the modified algorithm, we get a sharper image for a single tumor illustrated in Fig. 9(a,b). In the scenario of multiple tumors, DAS cant only detects a single tumor with ghosting around the surface in Fig. 9(c). We get a clearer image of two tumors on the right side for the corrected algorithm. The deviations appear twice over the transmitted S-parameter with low power artifact at about −0.02 and 0.02 positions. The artifact appears after 0.55 ns. The calculated time can be translated to distance using the propagation rates of signal in different media. Then the tumor is detected at roughly the same positions and a low power artifact at about 0.44 ns. In the time-domain output, the red color area indicates a higher reflection coefficient. Reflection caused due to the tumor increases the return loss resulting in the higher reflected power from the antennas to phantom.

Several metrics such as the accuracy, the localization error, the signal-to-clutter ratio (SCR), and the signal-to-mean ratio (SMR) of the reconstructed image are considered for evaluation criteria of an imaginmg detection system. In this article, we have considered SMR for the imaging accuracy analysis. The SMR is a measure of the quality of the beamformed image that provides a measure of separation between the reason of interest (ROI) and the background clutter. It is defined as the ratio of the average intensity of the ROI to the average intensity of the overall image. The SMR for two phantoms are listed in Table 2 for typical and proposed IC-DAS algorithm. The SMR describes the ratio of maximum backscattered energy from tumor to the mean energy response at the same sample. The SMR is considerably improved in two phantoms study. The comparison of proposed MWI to the existing systems is presented in Table 3. The proposed MWI system has some advantages over another system in terms of size, compactness, portability, cost-effectiveness. The proposed system exhibits better performance for breast phantom, which is promising for early-stage tumor detection.

**Table 2:** Signal to mean ratio.

Phantom	DAS	IC DAS
(1 tumor)	5.0645	5.0690
(2 tumors)	4.6943	4.6973

**Table 3:** Comparison of the different measurements system with the proposed system.

Ref.	Antenna Type	Operating Freq. GHz)	Elements/Position	Fixed/Movable	Frequency/Time domain	Imaging Method	Phantom And tumor object
^48^	Pyramidal Horn Antenna	2.0–10.0	8 × 241 Scanned position on transmission reception	Movable Multistatic	Frequency domain	No Results	No No
^49^	Balanced antipodal Vivaldi antenna	1.0–13.0	36 Single element scanned position	Fixed Tank Rotate monostatic	Frequency domain	TSAR (Tissue Sensing Adaptive Radar)	Sample Tissue No
^50^	Tapered and transmission loaded antenna	2.0–8.0	16 element Array 16 × 15 scanned position	Fixed Switching Matrix	Time-domain	DMAS (Delay Multiply and Sum Algorithm)	Lab-based breast phantom Yes Single
^51^	Slotted patch	3.5–15.0	4 × 4 Single element	Fixed Switching Matrix	Frequency domain	Confocal Imaging	Simulated phantom Yes
^52^	Corrugated Antipodal Vivaldi Antenna	1.0–4.0	16 Single element Scanned position	Fixed Rotated Platform Monostatic	Frequency domain	DAS Delay and Sum Algorithm)	Lab-based breast phantom No
^53^	UWB transceiver	3.0–10.0	16 Element array	Fixed Switching Matrix	Time-domain	DAS Simulation only	No No No
^54^	Slotted antipodal Vivaldi antenna	3.01–11.0	Two element 2 × 50 position	Fixed Platform rotated	Time-domain	DMAS	Yes Yes Single
^55^	CPW feed Monopole	2.0–4.0	16 Elements array 16 × 15 scanned position	Fixed Switching Matrix	Time-domain	DMAS	Yes Yes Single
^56^	CPW feed EBG structure Antenna	3.1–7.6	2 antenna elements 2 × 120 scanned position	Fixed Platform rotated	Frequency Domain	DMAS	Commercial phantoms Single tumor object
^57^	Horn like 3D UWB antenna	2.0–6.5	2 antenna elements 24 × 19 transmission-reception position	Movable multistatic	Time and Frequency Domain	DMAS	No phantom No tumor
Proposed	Side slotted Vivaldi antenna	2.80–7.00 GHz	9 Antenna array 8 × 50 scanned position	Movable multistatic	Frequency and Time Domain	IC-DAS	Lab-made heterogeneous phantom 1 and 2 tumor object

## Conclusion

This work is dedicated to the design, development, and implementation of a new, complete, portable, and UWB antenna based microwave imaging system that can be applied for breast tumor detection in real-time. A Side slotted UWB directional Vivaldi antenna is proposed where the antennas fulfill the requirements of microwave imaging because of their high directive nature with a high gain due to the tapered slot design and having a high peak value for the pulse envelope. The Vivaldi antenna also offers stable group delay and allows a narrow pulse width. To achieve these desired characteristics, a conventional Vivaldi antenna has been customized with the introduction of a number of slots on the radiating fins and named SSVA. Experimental validation of the antennas has been performed by developing a breast phantom measurement system to detect the unwanted tumor cells inside the breast. Several heterogeneous breast phantoms have been developed containing dielectric properties same as real breast tissue. A computer-controlled automatic mechanical approach is designed for microwave imaging system consists of a 1 × 9 antenna array, (one for transmitter and eight for receiver), the stepper motor based antenna mounting stand, the adjustable phantom hanging platform, a RF switching system to control the receivers and the personal computer-based signal processing and image reconstruction unit. The change of backscattered signals with the change of dielectric content inside the structure of breast phantom is analyzed. The significant variation of the back-scattered signal can exploit the unwanted tumor cells inside the breast. The collected data from the system are processed using the newly proposed IC-DAS imaging algorithm which reconstructs the image of the breast interior to detect even multiple tumors. By using the proposed side slotted Vivaldi antennas, the microwave imaging system can detect the tumor or unwanted cell inside the human breast.

## References

[1] Kahar M, Ray A, Sarkar D, Sarkar P (2015). An UWB microstrip monopole antenna for breast tumor detection. Microwave and Optical Technology Letters.

[2] Zhang, H. Microwave imaging for ultra-wideband antenna based cancer detection. (2015).

[3] Kuhl CK (2005). Mammography, breast ultrasound, and magnetic resonance imaging for surveillance of women at high familial risk for breast cancer. Journal of clinical oncology.

[4] Elmore JG (1998). Ten-year risk of false positive screening mammograms and clinical breast examinations. New England Journal of Medicine.

[5] Berg WA (2004). Diagnostic accuracy of mammography, clinical examination, US, and MR imaging in preoperative assessment of breast cancer. Radiology.

[6] Hassan AM, El-Shenawee M (2011). Review of electromagnetic techniques for breast cancer detection. IEEE Reviews in Biomedical Engineering.

[7] Bahrami, H. *et al*. In 2014 *36th Annual International Conference of the IEEE Engineering in Medicine and Biology Society*. 3775–3778 (IEEE). 10.1109/EMBC.2014.6944445 25570813

[8] Arnone D, Ciesla C, Pepper M (2000). Terahertz imaging comes into view. Physics World.

[9] Geetharamani G, Aathmanesan T (2019). Metamaterial inspired THz antenna for breast cancer detection. SN Applied Sciences.

[10] Guillet JP (2014). Review of terahertz tomography techniques. Journal of Infrared, Millimeter, and Terahertz Waves.

[11] Meaney PM, Fanning MW, Li D, Poplack SP, Paulsen KD (2000). A clinical prototype for active microwave imaging of the breast. IEEE Transactions on Microwave Theory and Techniques.

[12] Klemm M, Craddock IJ, Leendertz JA, Preece A, Benjamin R (2009). Radar-based breast cancer detection using a hemispherical antenna array—experimental results. IEEE Transactions on Antennas and Propagation.

[13] Semenov S (2011). Microwave tomography of extremities: 1. Dedicated 2D system and physiological signatures. Physics in medicine and biology.

[14] Semenov S (2011). Microwave tomography of extremities: 2. Functional fused imaging of flow reduction and simulated compartment syndrome. Physics in medicine and biology.

[15] Fear EC (2013). Microwave breast imaging with a monostatic radar-based system: A study of application to patients. IEEE transactions on microwave theory and techniques.

[16] Porter E, Santorelli A, Popovic M (2014). Time-domain microwave radar applied to breast imaging: Measurement reliability in a clinical setting. Progress In Electromagnetics Research.

[17] Amineh RK, Ravan M, Trehan A, Nikolova NK (2011). Near-field microwave imaging based on aperture raster scanning with TEM horn antennas. IEEE Transactions on Antennas and Propagation.

[18] Lazaro A, Girbau D, Villarino R (2009). Simulated and experimental investigation of microwave imaging using UWB. Progress In Electromagnetics Research.

[19] Mohammed BAJ, Abbosh AM, Sharpe P (2013). Planar array of corrugated tapered slot antennas for ultrawideband biomedical microwave imaging system. International Journal of RF and Microwave Computer-Aided Engineering.

[20] Zhang D, Mase A (2011). Experimental study on radar-based breast cancer detection using UWB antennas without background subtraction. Biomedical Engineering: Applications, Basis and Communications.

[21] Islam MM (2015). A miniaturized antenna with negative index metamaterial based on modified SRR and CLS unit cell for UWB microwave imaging applications. Materials.

[22] Zhang J, Fear EC, Johnston RH (2009). Cross-Vivaldi antenna for breast tumor detection. Microwave Opt. Technol. Lett.

[23] Islam MM (2015). Microwave imaging sensor using compact metamaterial UWB antenna with a high correlation factor. Materials.

[24] Jafari H, Deen J, Hranilovic S, Nikolova N (2007). Co-polarised and cross-polarised antenna arrays for breast, cancer detection. Microwaves, Antennas & Propagation, IET.

[25] Kurra L, Abegaonkar MP, Basu A, Koul SK (2016). FSS Properties of a Uniplanar EBG and Its Application in Directivity Enhancement of a Microstrip Antenna. IEEE Antennas and Wireless Propagation Letters.

[26] Langley RJ, Parker EA (1983). Double-square frequency-selective surfaces and their equivalent circuit. Electronics Letters.

[27] Alzabidi, M. A., Aldhaeebi, M. A. & Elshafiey, I. In *Electronics*, *Communications and Photonics Conference (SIECPC)*, 2013 *Saudi International*. 1–4 (IEEE).

[28] De Oliveira AM, Perotoni MB, Kofuji ST, Justo JF (2015). A palm tree antipodal Vivaldi antenna with exponential slot edge for improved radiation pattern. IEEE Antennas and Wireless Propagation Letters.

[29] Nassar IT, Weller TM (2015). A Novel Method for Improving Antipodal Vivaldi Antenna Performance. Antennas and Propagation, IEEE Transactions on.

[30] Abbak M, Çayören M, Akduman I (2014). Microwave breast phantom measurements with a cavity-backed Vivaldi antenna. IET Microwaves, Antennas & Propagation.

[31] Wu, B., Ji, Y. & Fang, G. In *Electronic Measurement & Instruments*, *ICEMI'09*. *9th International Conference on*. 2-226-222-229 (IEEE) (2009).

[32] He SH (2014). An improved vivaldi antenna for vehicular wireless communication systems. Antennas and Wireless Propagation Letters, IEEE.

[33] Pandey G, Verma H, Meshram M (2015). Compact antipodal Vivaldi antenna for UWB applications. Electronics Letters.

[34] Hu S, Dou W, Law C (2009). A tapered slot antenna with flat and high gain for ultra-wideband applications. Journal of Electromagnetic Waves and Applications.

[35] Fear EC, Meaney PM, Stuchly MA (2003). Microwaves for breast cancer detection?. IEEE potentials.

[36] Kanj H, Popovic M (2005). Miniaturized microstrip-fed” Dark Eyes” antenna for near-field microwave sensing. IEEE Antennas and Wireless Propagation Letters.

[37] Guillanton E, Dauvignac J, Pichot C, Cashman J (1998). A new design tapered slot antenna for ultra-wideband applications. Microwave and Optical Technology Letters.

[38] Chiappe M, Gragnani GL (2006). Vivaldi antennas for microwave imaging: Theoretical analysis and design considerations. Instrumentation and Measurement, IEEE Transactions on.

[39] Abu Bakar A, Ireland D, Abbosh AM, Wang Y (2012). Experimental assessment of microwave diagnostic tool for ultra-wideband breast cancer detection. Progress In Electromagnetics Research M.

[40] Fear EC, Meaney PM, Stuchly M (2003). Microwaves for breast cancer detection? Potentials. IEEE.

[41] Lu J-H, Tsai F-C (2013). Planar internal LTE/WWAN monopole antenna for tablet computer application. Antennas and Propagation, IEEE Transactions on.

[42] Islam MS, Kibria S, Islam MT (2018). Experimental Breast Phantoms for Estimation of Breast Tumor Using Microwave Imaging Systems. IEEE Access.

[43] Gibson, P. In *Microwave Conference*, *9th European*. 101–105 (IEEE) (1979).

[44] Natarajan R (2016). Modified antipodal Vivaldi antenna for ultra-wideband communications. IET Microwaves, Antennas & Propagation.

[45] Klemm, M., Craddock, I., Leendertz, J., Preece, A. & Benjamin, R. Improved delay-and-sum beamforming algorithm for breast cancer detection. *Int J Antennas Propag* **2008** (2008).

[46] Kibria, S. *et al*. Breast Phantom Imaging using Iteratively Corrected Coherence Factor Delay and Sum. *IEEE Access* (2019).

[47] Elahi M (2018). Evaluation of Image reconstruction algorithms for confocal microwave imaging: Application to patient data. Sensors.

[48] Geffrin J-M, Sabouroux P, Eyraud C (2005). Free space experimental scattering database continuation: experimental set-up and measurement precision. inverse Problems.

[49] Salvador SM, Fear EC, Okoniewski M, Matyas JR (2010). Exploring joint tissues with microwave imaging. IEEE Transactions on Microwave Theory and Techniques.

[50] Porter E, Kirshin E, Santorelli A, Coates M, Popovic M (2013). Time-domain multistatic radar system for microwave breast screening. IEEE Antennas Wireless Propag. Lett.

[51] Sugitani T, Kubota S, Toya AXX, Kikkawa T (2013). A Compact 4 × 4 Planar UWB Antenna Array for 3-D Breast Cancer Detection. IEEE Antennas Wirel. Propag. Lett..

[52] Beada’a JM, Abbosh AM, Mustafa S, Ireland D (2014). Microwave system for head imaging. IEEE Trans. Instrum. Meas..

[53] Kwon S, Lee SJEL (2013). Instantaneous microwave imaging with time-domain measurements for breast cancer detection. Electronics Letters.

[54] Islam M, Samsuzzaman M, Islam M, Kibria S, Singh MJS (2018). A homogeneous breast phantom measurement system with an improved modified microwave imaging antenna sensor. sensors.

[55] Porter E (2016). A wearable microwave antenna array for time-domain breast tumor screening. IEEE transactions on medical imaging.

[56] Mahmud MZ, Islam MT, Misran N, Kibria S, Samsuzzaman M (2018). Microwave Imaging for Breast Tumor Detection Using Uniplanar AMC Based CPW-Fed Microstrip Antenna. IEEE Access.

[57] Shao W, Edalati A, McCollough TR, McCollough WJ (2018). A Time-Domain Measurement System for UWB Microwave Imaging. IEEE Transactions on Microwave Theory and Techniques.

